# Compound Presentation

**Published:** 1889-11

**Authors:** R. H. L. Bibb

**Affiliations:** Saltillo, Mexico


DANIEL'S

### Texas Medical Journal.

A Representative Organ of the Medical Profession, and an Exponent of Rational Medicine; devoted to the Organization, Advancement and Elevation of the Profession in Texas.

Published Monthly.—Subscription $2.00 a Year.

Vol. V. AUSTIN, NOVEMBER, 1889. No. 5.

### Original Articles.

CONTRIBUTED EXCLUSIVELY TO THIS JOURNAL.

*The Articles in this Department are accepted and published with the understanding that we are not responsible for, nor do we indorse the views and opinions of the writers by so doing.*

For Daniel's Texas Medical Journal.

### COMPOUND PRESENTATION.

BY. R. H. L. BIBB, M. D., SALTILLO, MEXICO.

MRS. P., a small, healthy multipara, thirty years of age, expected her fifth confinement about the first of Ootober, and engaged my services for the occasion.

On the sixteenth of September, I was summoned, very hurriedly, to visit Mrs. P. I found her in bed, very greatly agitated, and learned from her that a half hour previous to my arrival, while standing, bending over a table, she felt something give way and that the sensation was accompanied by the discharge of an enormous quantity of water—that she was deluged. She said she felt but little pain at the time, and but little since, and that, aside from the exceptional—with her—distension of the abdomen—which she claimed to have been twice as large as with her former pregnancies—and its attending inconveniences, nothing unusual had attracted her attention during the present gestation. Her prevenient accouchements had been normal in every respect.

On making a digital examination—I presumed there had been rupture of the amnion and escape of its fluid—I found one hand, left, and a portion of the funis, in the vagina; the head,—in the fifth, Leisman's fourth, cranial position,—the right hand and the left foot, presenting at the partially, about one third, dilated os.

My old friend and confrere, Dr. Smith, being close at hand, and wishing to avail myself of his experience and assistance in the management of the case, his presence was requested. The doctor agreed with me, that the parts were presenting as detailed above; concurred in the opinion of the necessity of turning, and kindly consented to administer chloroform, while I should make version. With this end in view, the patient chloroformed, placed in the dorsal position, and observing as closely as possible, the principles of antiseptic midwifery so strenuously insisted on by Garrigues* I introduced my right hand, pushed the head up towards the mother's left side, siezed the presenting foot and attempted to turn by making traction upon it, aided by external manipulation. Failing in this, and fearing to make greater traction force than that already applied, I passed a running noose of tape around the ankle of the presenting foot, in order not to displace it, and sought for, and caught hold of the other foot. With this in hand, during complete uterine relaxation, version was easily accomplished by bringing the right foot down by the side of, and out at, the vulva with its fellow, thus converting a complicated fifth cranial position into something like a second breech position.

I now requested my assistant to discontinue the anæsthetic—it had not been pushed much beyond so-called obstetric anesthesia

*Amer: Sys: Obstet: Vol. 2.

—and to make firm pressure over the uterine fundus, and to follow the head down closely, as I extracted the fœtus. This the doctor did so intelligently and so successfully, that I had no trouble in delivering the hips, body, shoulders, and arms in rapid succession. The child was now gasping, and the cord pulseless—it had been drawn down and protected as far as possible—so I quickly passed the palmar aspect of the index and middle fingers of my left hand, along the front of the child's neck, up over the chin, into its mouth, and made traction downwards, by gently pulling with the fingers in the mouth, while with my right hand, I expressed the head out of the pelvis, as it were, by grasping hold of the occiput through the abdominal—as in the Wigand-Martin method—in the direction of axis of the superior strait,* thus bringing to a happy termination, for both mother and child, a unique, and a highly interesting case of midwifery. As there was neither cupping of the fundus, uterine tetanus, cervical obstruction, nor impaction of the shoulder,—the chief impediments to version,—and as my efforts to turn were made with considerable force, during uterine relaxation and under chloroform, I am at a loss to account for the failure of my first attempt. The placenta, a large one, was attached to the right antero-lateral half of the uterus, but in my humble opinion presented no obstacle to version.

It will be observed that I have had the temerity to designate as unique the case herein recorded. This was not done in the belief that no such case had been observed by others; but for the reason that with me the case was unique, and for the further reason that I have never seen a case reported presenting the complications of the one to which I here call attention. Leishman (†) reports having met with a case where "the head, hand, foot and cord," presented, and La Motte (‡) relates one where "the

*I have used this method on various occasions, with like happy results, and I commend it, most heartily, to those having to deal with 'head last' cases.

(†) Sys. of Midwifery, p. 354.

(‡) Quoted by Parwin, Amer. Sys. Obstet., vol. 1, p. 758.

head, the hands and one of the feet presented." This case differs from Leishman's in that both hands presented, and from La Motte's in the prolapse of the cord.

Parvin (§) says, "all authors who have written upon this subject * * have admitted as predisposing or occassional causes" of compound presentations, the small size of the fœtus, the abundance of the amniotic liquor, its rapid discharge, oblique presentation of the fœtus, and, finally, vices of conformation of the pelvis:" He then goes on to say, that "Charpentier adds to these causes rupture of the membranes, while the woman is standing, and unskilful or untimely efforts to perform version."

In this case the child weighed seven pounds; there was no vice of conformation of the pelvis; there was no unskilful or untimely efforts to perform version; but there was an abundance of amniotic liquor, and its rapid discharge, while the patient was standing, benidng over a table, hence, I conclude that the latter had more to do than the oblique presentation of the fœtus did, with producing the compound presentation described above.

NOTE.—Since the above was written, my attention has been called to a case reported by Dr. Paine, of Galveston, (Trans. T. S. M. A., 1885, p. 361), where "the face, both hands, one foot, and cord" presented.

(§) Amer. Sys.Obstet., vol. 1, p. 758.

